# Gastric Medullary Carcinoma: A Rare Case Report

**DOI:** 10.1155/2016/2875471

**Published:** 2016-08-03

**Authors:** Ferit Aslan, Fisun Ardıç Yükrük, Fatma Buğdaycı Başal, Ayşe Durnalı

**Affiliations:** ^1^Medical Oncology Department, Dr. Abdurrahman Yurtaslan Oncology Training and Research Hospital, Mehmet Akif Ersoy Quarter 13, Street No. 56, Yenimahalle, 06200 Ankara, Turkey; ^2^Pathology Department, Dr. Abdurrahman Yurtaslan Oncology Training and Research Hospital, Mehmet Akif Ersoy Quarter 13, Street No. 56, Yenimahalle, 06200 Ankara, Turkey

## Abstract

A case of 64-year-old female patient with early stage gastric medullary carcinoma has been presented, along with a review of the literature.

## 1. Introduction

Gastric medullary carcinoma (GMC) is a rare neoplasm characterized by dense lymphocytic infiltration of the stroma. It is more frequent after the 6th decade of life in males and is generally considered to have a moderate prognosis. GMC has been associated with either microsatellite instability (MSI) or the Epstein-Barr virus (EBV). Herein, we present a case of early stage GMC.

## 2. Case Report

**Figure 1:**
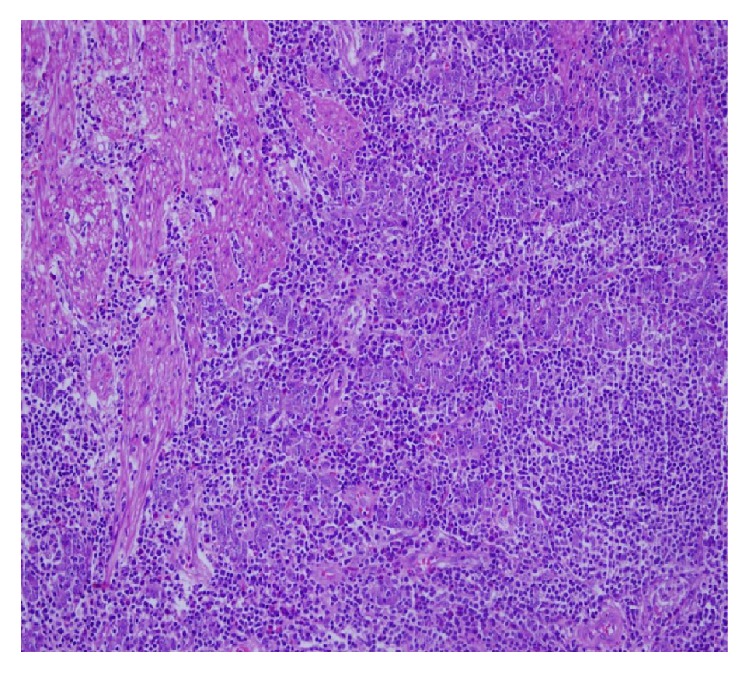
Muscular layer infiltration of atypical cells formed syncytial islands, HEx200.

A 64-year-old female patient with an otherwise normal medical history presented with abdominal pain over the previous four months. An upper gastrointestinal (GI) endoscopy showed an ulcerative lesion on the cardia of the stomach, and the pathological findings revealed a signet-ring cell adenocarcinoma of the stomach. The patient underwent a total gastrectomy, and the postoperative histological findings were consistent with medullary carcinoma with a lymphoid stroma. The surgical margins and extracted lymph nodes were negative. The pathological specimens, which were reexamined in our institute in order to confirm the diagnosis, were compatible with prominent lymphoid cell infiltration without evidence of signet-ring cells (Figure 1). The disease was staged as a T2N0M0 GMC and adjuvant capecitabine chemotherapy was planned. This patient had no relapse through 25 months of follow-up.

**Figure 2:**
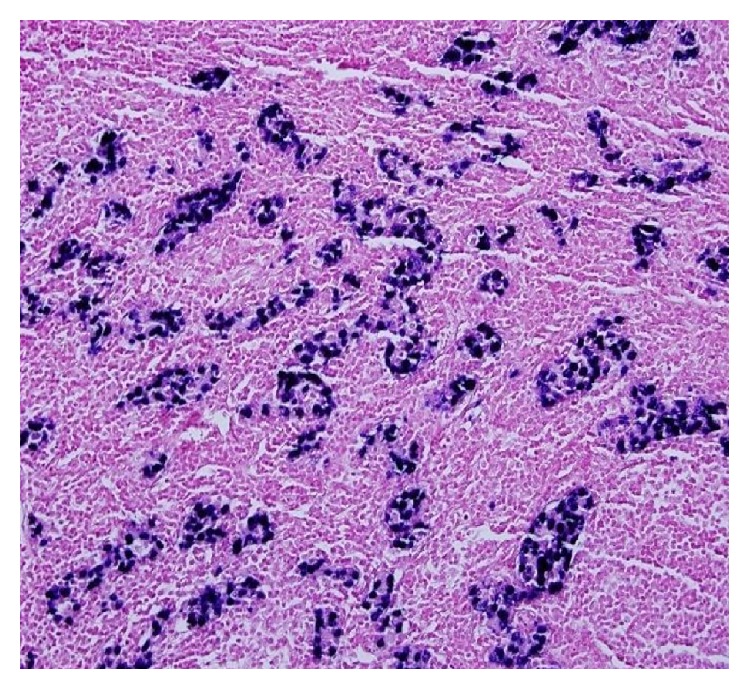
In situ hybridization studies for EBER test showed diffuse positive staining in the tumour cells.

The tumour tissue specimens were also examined to investigate any possible associations with MSI and EBV. Positive staining for MLH1, MSH2, PMS2, and MSH6 was observed in the immunohistochemical analyses of the neoplastic cells; however, no significant loss of nuclear expression was present. The in situ hybridization studies using the EBV-encoded small RNA (EBER) test showed diffuse positive staining in the tumour cells (Figure 2).

## 3. Discussion

GMC is a rare disease that is commonly seen in males over sixty years of age [1], and it is usually diagnosed in the early stages [2]. The World Health Organization (WHO) uses the terms medullary carcinoma and lymphoepithelioma-like carcinoma as synonyms for gastric carcinoma with lymphoid stroma [3]. Undifferentiated gastric carcinoma with lymphoid stroma is characterized microscopically by the attachment of neoplastic epithelial cells that show intensive lymphoid proliferation [4]. This stromal lymphoid infiltration generally involves T and B lymphocytes, multinucleated giant macrophages, and dendritic and plasma cells [5].

Although the WHO considers GMC to be synonymous with lymphoepithelioma-like carcinoma, Chetty has determined that gastric medullary carcinoma and lymphoepithelioma-like carcinoma differ from each other. Medullary carcinoma is frequently associated with MSI, and lymphoepithelioma-like carcinoma is associated with EBV [6]. The lymphocytes in medullary carcinoma aggregate in syncytial islets and at the peripheral margins, while the inflammation is peritumoural. Lymphocytes show continuity at the tumoural margins in lymphoepithelioma-like carcinoma, and they are localized as a wide number of intratumoural lymphocytes, instead of small aggregates [6].

Burke et al. have indicated an association between gastric lymphoepithelioma-like carcinoma and the EBV [7]. In 1994, Takano et al. subsequently reported an EBV-associated GMC with lymphoid infiltration [8]. Grogg and his team reported that EBV and MSI exclude each other in lymphocyte-rich gastric cancer [9], which was similar to our case, in which the MSI was not high in any of the tumours that showed EBV positivity [6, 9].

GMC differs in some of its clinicopathological properties from a well-differentiated adenocarcinoma; however, the biological behaviour and duration of survival of these two tumours are similar [10]. Medullary carcinoma exists in a smaller area and is less invasive; moreover, it is less likely to originate in the upper regions of the stomach, when compared with adenocarcinoma [10]. EBV-associated GMC with lymphocyte infiltration is 90% localized in the cardia and corpus [8]. Our case was diagnosed as GMC with a lymphoid stroma, and the tumour was localized in the cardia; it was a T2 tumour and was not deeply invasive. Venous invasion is relatively common in both tumours, and hematogenous liver metastases are the most frequent reasons for death. The relationship between GMC and angiogenesis has been investigated, and correlations have been determined between the number of vessels, expression of the vascular endothelial growth factor, and stage of the disease [11].

In summary, although it is rare, GMC is an interesting carcinoma with regard to its association with EBV or MSI. Early diagnosis, the course of the disease with regard to hematogenous metastases, and experience with previous cases (despite the similarity with well-differentiated adenocarcinoma) are among the factors that affect a patient's treatment and outcome.
